# Body fat measurement by bioelectrical impedance and air displacement plethysmography: a cross-validation study to design bioelectrical impedance equations in Mexican adults

**DOI:** 10.1186/1475-2891-6-18

**Published:** 2007-08-15

**Authors:** Nayeli Macias, Heliodoro Alemán-Mateo, Julián Esparza-Romero, Mauro E Valencia

**Affiliations:** 1 Centro de Investigación en Nutrición y Salud, Instituto Nacional de Salud Pública. Universidad 655, Santa Maria Ahuacatitlan, CP 62508, Cuernavaca, México; 2 División de Nutrición, Centro de Investigación en Alimentación y Desarrollo, A.C. Carretera a la Victoria km, 0.6 Hermosillo, Sonora, Apartado Postal 1735, CP 8300, México

## Abstract

**Background:**

The study of body composition in specific populations by techniques such as bio-impedance analysis (BIA) requires validation based on standard reference methods. The aim of this study was to develop and cross-validate a predictive equation for bioelectrical impedance using air displacement plethysmography (ADP) as standard method to measure body composition in Mexican adult men and women.

**Methods:**

This study included 155 male and female subjects from northern Mexico, 20–50 years of age, from low, middle, and upper income levels. Body composition was measured by ADP. Body weight (BW, kg) and height (Ht, cm) were obtained by standard anthropometric techniques. Resistance, R (ohms) and reactance, Xc (ohms) were also measured. A random-split method was used to obtain two samples: one was used to derive the equation by the "all possible regressions" procedure and was cross-validated in the other sample to test predicted versus measured values of fat-free mass (FFM).

**Results and Discussion:**

The final model was: FFM (kg) = 0.7374 * (Ht^2 ^/R) + 0.1763 * (BW) - 0.1773 * (Age) + 0.1198 * (Xc) - 2.4658. R^2 ^was 0.97; the square root of the mean square error (SRMSE) was 1.99 kg, and the pure error (PE) was 2.96. There was no difference between FFM predicted by the new equation (48.57 ± 10.9 kg) and that measured by ADP (48.43 ± 11.3 kg). The new equation did not differ from the line of identity, had a high R^2 ^and a low SRMSE, and showed no significant bias (0.87 ± 2.84 kg).

**Conclusion:**

The new bioelectrical impedance equation based on the two-compartment model (2C) was accurate, precise, and free of bias. This equation can be used to assess body composition and nutritional status in populations similar in anthropometric and physical characteristics to this sample.

## Background

The importance of measuring body composition has increased, due to the need to evaluate changes in nutritional status, which can affect body reserves differentially. Subjects can gain or lose body fat, fat-free mass, bone mineral mass or cellular mass components as a result of disease, overeating, sports, or undernutrition (anorexia nervosa), or as a result of nutritional intervention programs. These changes can only be detected by using valid body composition measurement techniques. In addition, the world-wide epidemic of obesity and its association with chronic disease has also contributed to the need to study body composition [1,2]and the distribution of body components [3]. The definition of obesity based on body size has been challenged in regard to different populations, due to the different relationships of percent body fat at the same BMI levels [4]. Therefore, a definition of obesity based on body composition terms can help to clarify this issue.

In order to have good body composition estimates from bioelectrical impedance techniques, it is necessary to have proper validation based on reference methods of better accuracy and precision. Recently, it has been reported that air displacement plethysmography (ADP) is a valid method to determine body composition compared with hydrodensitometry and the four compartment model in adults [5,6]. However, these standard reference methods are difficult to implement in some clinical settings and in epidemiological studies.

It is well recognized that the BIA technique when properly validated is a practical option for these types of studies. Body composition assessment by bioelectrical impedance can be improved when the specific equations used are validated against reference methods and for a specific population [7,8]. Furthermore, validated BIA equations can overcome some limitations due to differences in fat distribution patterns and limb length (arm and leg) [9] between ethnic groups that might affect the accuracy and precision of the technique [10]. Finally, it is important to mention that most published BIA equations derive from Caucasian populations, and presently there are no published equations for Mexican adults. The aim of this study was to develop and cross-validate a predictive equation for bioelectrical impedance using air displacement plethysmography (ADP) as a standard method to measure body composition in Mexican adult men and women.

## Methods

This was a cross-sectional study in healthy men and women, 20 to 50 years of age, residing in Sonora, Mexico. Participants were selected by nonprobabilistic sampling between 2000 and 2001. Pregnant and lactating women and subjects with diabetes, cardiovascular disease, or any other condition that could cause hydro-electrolytic imbalance were excluded. In addition, none of the participants were on diuretics or other medication that could alter body composition. The Ethics Committee of the Centro de Investigacion en Alimentacion y Desarrollo (CIAD: Food and Development Research Center) approved the study.

All body composition measurements were performed in CIAD's metabolic unit. Prior to the measurements, all participants signed a written consent form after being informed of procedures and purpose of the study. Subjects were asked to fast for 12 h, to not perform exercise the day before the measurements, and to empty their bladder prior to evaluation.

Human body volume, body density, and body composition were evaluated with ADP (Bod Pod, Body Composition System, Life Measurement Instruments, Concord, CA). The system determines body volume by the application of Boyle's gas law. The ADP unit consists of a dual-chamber plethysmograph, an electronic scale, and a computer. This equipment has a single structure containing two chambers separated by a device that produces pressure fluctuations and volume changes that permit the assessment of body volume. The system has been described in detail elsewhere [11]. Wearing a swimsuit and acrylic swimming cap, subjects were first weighed to the nearest 0.01 kg with the ADP electronic scale. A two-point calibration was performed, with the chamber empty and with a 50 L cylinder. After the calibration procedure, the subject's body volume was measured twice while they were seated quietly in the test chamber and breathing normally. In the second trial, thoracic gas volume was measured to correct the body volume. From the corrected body volume and body mass values, body density was obtained and percent body fat was calculated using Siri's equation and ADP software. Fat-free mass was calculated from the two-compartment model [12]. All measurements were done following the manufacturer's instructions.

The reproducibility of air displacement plethysmography system was tested using duplicate measurements in 91 subjects. The duplicate measurements were both performed during the same session. The duplicated mean body density measurements were 1.0292 ± 0.019 and 1.0296 ± 0.019, and the mean difference in body density was 0.00007. The coefficient of variation for both measurements was 1.89%. In terms of %BF, the technical error of measurement was 0.066 % fat units, assessed as (Σd2/2n)1/2, where d is the difference between repeated measurements and n is the number of paired repeated measurements.

Anthropometry was performed by a standardized technician according to Lohman's recommendations [13]. Body weight (BW) was measured in subjects dressed in a swimsuit using a digital electronic scale (150 ± 0.01 kg) connected to the ADP. Standing height was measured with a Holtain stadiometer to the nearest millimeter (205 ± 0.5 cm, Holtain Limited, Dyfed, UK). Body mass index (BMI, kg/m^2^) was calculated based on weight and standing height. Waist circumference was measured in supine position at the umbilicus level, and hip circumference was evaluated in standing position at the level of the most prominent part of the gluteus. Both measurements were done with a fiberglass measuring tape (Lafayette Instruments Company Inc., USA). Waist/hip ratio (WHR) was determined from these measurements.

Resistance (R) and reactance (Xc) were measured with a bioelectrical impedance analyzer (Model BIA-103, RJL Systems Detroit, MI) according to the manufacturer's instructions. BIA equipment calibration was periodically performed with an electrical resistor. Calibration values were considered normal if they were not higher than 498 ± 2 ohms (Ω). Volunteers were instructed to lie supine with their hands at their side and with their legs separated. The skin surface was cleaned with ethanol, and the electrodes were placed on the dorsum of the right foot and hand. All measurements were performed according to Lukasky [14].

Data were analyzed using the statistical program, NCSS 2001 (Number Cruncher Statistical System for Windows, Kaysville, Utah). The *t*-test for independent samples was used to evaluate differences of general characteristics between men and women and also between samples. All results are expressed as means ± standard deviations (SD).

For the design of the BIA equation, the total sample (*n *= 155) was used in a split-sample internal cross-validation. In this approach, the sample was split randomly into subsamples of approximately the same size (78 and 77 subjects each) (Table 2). The regression equation was developed in a randomized sample of 78 subjects. This equation had the lowest SEE and highest R^2^, and was cross-validated using the second sample of 77 subjects. Model selection was carried out using the "all possible regressions" procedure. This method guarantees finding the model having the largest R^2 ^and the smallest square root of MSE (SRMSE). Mallow's Cp statistic was used to optimize model selection. Multiple regression procedure was used to analyze the relation between FFM as a dependent variable with: age (years), sex (male = 1, female = 0), body weight (kg), square height (cm)/resistance (Ω) (Ht^2^/R), and reactance (Ω) as independent variables. Multicolinearity was analyzed by regression diagnostics using the condition number (CN < 30) and the variance inflation factor (VIF < 10). The pure error was calculated as the square root of the sum of squared differences between the observed and the predicted values divided by the number of subjects in the cross-validation sample [15].

Validation of the new BIA equation was done using the split-sample internal cross-validation method. Body composition was estimated in the second randomized group or validation sample (77 subjects) using the new equation developed from the first randomized group, or equation sample (78 subjects). Estimates of body composition, particularly the FFM predicted by the new BIA equation, were compared to values measured by ADP (the reference method) using a paired *t*-test. The accuracy and precision of the new BIA equation were tested by regression procedures. It was considered accurate if the regression between fat-free mass by ADP and the new BIA equation had a slope not significantly different from 1.0 and an intercept not significantly different from zero. Precision was assessed by the model R^2 ^and the standard error of the estimate from the regression procedures described above. Bias was examined using Bland and Altman's analysis [16] and regression procedures.

## Results

In this study, 155 healthy adults (82 women and 73 men) were evaluated. General characteristics of the subjects are presented in Table 1. As expected, the men had significantly higher values of weight, height, waist, and waist/hip ratio compared with the women.

**Table 1:** General characteristics of the study group.

	***Female (n = 82)***		***Male (n = 73)***	
	Mean ± SD	Range	Mean ± SD	Range
Weight (kg)	65.7 * ± 12. 9	42.9–99.0	79.0 * ± 13.1	51.3–121
Age (years)	34.3 ± 7.6	22–48	33.9 ± 7.3	21–47
Height (m)	1.61* ± 0.05	1.45–1.80	1.73 * ± 0.05	1.58–1.8
BMI (kg/m^2^)	25.4 ± 4.4	18.2–35.5	26.2 ± 3.6	17.8–35.3
Waist	83.4* ± 12.0	60–116	91.8* ± 10.5	69–120
WHR	0.81* ± 0.08	0.63–1.1	0.90* ± 0.05	0.78–1.1

*: significant differences, p < 0.05. WHR: Waist to hip ratio

Table 2 shows the main variables in the randomly split samples. There were no significant differences between the values from the randomized sample used for developing the equation and the randomized sample for testing it. The equation obtained was:

**Table 2:** Some general characteristics of subjects from the split sample^a^.

	***Equation Sample (n = 78)***	***Validation Sample (n = 77)***	***p***
Weight (kg)	72.7 ± 15.3	71.3 ± 13.9	0.56
Age (years)	34.3 ± 7.4	34.0 ± 7.6	0.84
Height (m)	1.67 ± 0.09	1.66 ± 0.08	0.74
BMI (kg/m^2^)	25.9 ± 3.8	25.6 ± 4.2	0.68
R (ohms)	551 ± 81.4	552 ± 97.9	0.06
Xc (ohms)	57.6 ± 7.06	58.0 ± 10.3	0.79
H^2^(cm)/R (ohms)	52.2 ± 11.9	52.1 ± 11.7	0.96
BF (%)^b^	31.4 ± 7.9	31.5 ± 8.9	0.94
FFM (kg)^b^	49.7 ± 11.0	48.6 ± 10.2	0.52

^a^Mean ± SD. ^b ^ADP: Air displacement plethysmography; R: resistance; X_C_: reactance

FFM (kg) = 0.7374 * (Ht^2^/R) + 0.1763 * (BW)- 0.1773 * (Age) + 0.1198 * (Xc)- *2.4658*

Where: Ht^2 ^is height in cm, R is resistance in ohms, BW is body weight in kg, age is in years, and Xc is reactance in ohms

The independent variables that most accurately estimated fat-free mass were Ht2/R, BW, Xc, and age (Table 3). By partial R2 analysis, Ht2/R and body weight were the best individual predictors of FFM (kg), and accounted for 91.5% and 72.7% respectively, whereas reactance accounted for 2.56% and age for only 0.38% (Table 3). The final model included all these variables, with an F value of 568, R^2 ^of 0.97, an SRMSE of 1.99 kg, and a pure error (PE) of 2.96. Sex was not selected in this model as an independent variable. No multicolinearity was detected according to the regression diagnostic; the variance inflation factor (VIF) was 7.7 and the condition number (CN) was 9.82.

**Table 3:** Prediction equation for fat-free mass (n = 78).

	***Developed equation***					***Cross-Validated equation***			
	
	Intercept	Age	H^2^/R	BW(kg)	Xc (Ω)	R^2^	MSE (kg)	SQRRMSE (kg)	Pure Error
	**- 2.4658**	**- 0.1773**	**0.7374**	**0.1763**	**0.1198**	**0.969**	**3.97**	**1.99**	**2.96**
R^2 ^increase when this IV is added	-	0.0038	0.9323	0.0281	0.0047	-	-	-	-
Total R^2^ for this IV and the rest	-	0.0038	0.9361	0.9642	0.9689	-	-	-	-
R^2 ^when this IV is fit alone	-	0.0038	0.9149	0.7273	0.0256	-	-	-	-
Partial R^2 ^adjusted for other IVs	-	0.2920	0.8767	0.420	0.1303	-	-	-	-

Ht^2^/R = Height^2 ^(cm)/resistance (Ω); BW = Body weight (kg); Xc = reactance (Ω); MSE = Mean square error, SQRMSE = Square root of MSE; IV = independent variable.

The use of the paired *t*-test showed that there was no significant difference between the FFM measured with ADP and that estimated with the new BIA equation validated in the present study (Table 4) (p < 0.05). Furthermore, the percent-body-fat values obtained with the two compartment model from the new, validated BIA equation did not differ from the percent-body-fat values measured by ADP.

**Table 4:** Body fat (%) and fat free mass (kg) by different methods (mean ± SD).

	***Males (n = 73)***		***Females (n = 82)***	
	***Body Fat (%)***	***Fat Free Mass (kg)***	***Body Fat (%)***	***Fat Free Mass(kg*)**

ADP ^a^	25.9 ± 6.9	57.9 ± 7.1	36.2 ± 6.5	41.4 ± 6.2
BIA ^b^	25.8 ± 5.9	57.9 ± 6.5	36.1 ± 6.1	41.4 ± 5.9

^a ^ADP: air displacement plethysmography, reference method. ^b ^Body composition values calculated with the BIA equation validated in the present study.

**Figure 1:**
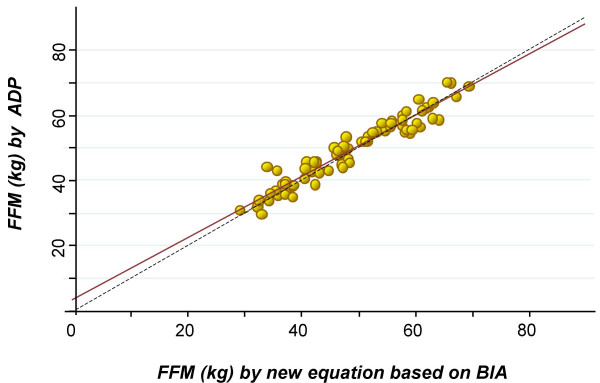
Regression between fat free mass by ADP and the new equation based on BIA.

**Figure 2:**
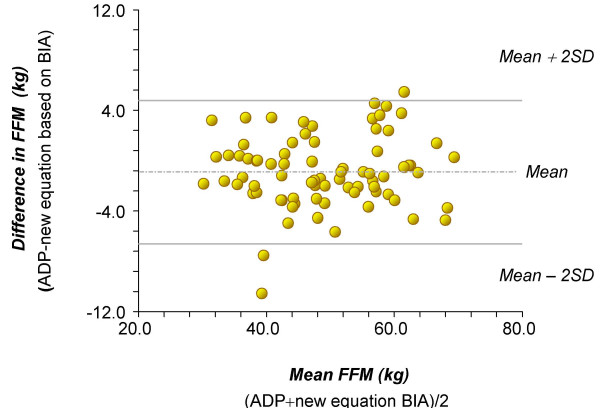
Bland and Altman analysis.

The mean values of FFM predicted by the new BIA equation and measured by ADP in the validation sample were 48.57 ± 10.9 kg and 48.43 ± 11.3 kg, respectively. There was no significant difference from the line of identity: the intercept was 0.02 and not different from zero, and the slope was 0.98 and not different from 1.0. The R^2 ^was 0.92 and the SRMSE was 2.86 (Figure 1). Bland and Altman analysis revealed that the bias expressed as the mean of the difference in FFM measured by ADP and that estimated from the new BIA equation was -0.87 ± 2.84 kg of FFM. The limits of agreement, defined as mean ± 2 SDs, were from -6.56 to + 4.82 kg of FFM (Figure 2). Furthermore, there was no significant association (p > 0.05) between the mean differences and the mean of two measurements (FFM obtained by ADP and the new validated equation (Figure 2).

## Discussion

The equation developed in the present study is the first validated BIA equation for Mexican adults, to the authors' knowledge. We found the new equation to be accurate, precise, and free of bias. FFM, and therefore FM, values obtained with equations developed for specific populations are more accurate and precise [8], as seen in the present study. The equation developed in this study takes into account the physical and body composition characteristics of the group used to design the equation, making it more population/environment specific.

Ethnicity has been shown to be an important factor when looking at the relationships of percent body fat and BMI. Among Chinese, Malay and Indian subjects, the Chinese showed the lowest body-fat value and Indians the highest at the same BMI level [17]. In our study, the BF% by the reference method was 25.9 in men and 36.2 in women at BMI values of 25.4 and 26.2, respectively. The percent body fat for the Mexican subjects of this study seems to be more similar to that of the Asian population reported by Gallagher [4] than to that obtained from Caucasians and Afro-Americans, who have less body fat at the same BMI. In addition to the differences in BMI, other researchers have reported fat distribution discrepancies between ethnic groups that could diminish the accuracy of body composition values obtained with BIA and anthropometric equations [18-21].

Casas et al. [21], evaluated 54 Hispanic women and 56 white women to determine whether Hispanic ethnicity is associated with total and regional adiposity and low FFM in healthy women. They found that Hispanic women have higher adiposity levels than white women. This difference reflected a higher amount of fat in the trunk. Further, abdominal and subscapular thicknesses were greater in Hispanic than in white women. These findings support the need to develop validated BIA and anthropometric equations for Hispanics. The best BIA equations developed for specific population groups have been properly validated [22-24].

The body composition variables selected in the final model of this study have been reported as the best predictors in BIA equations designed to estimate FFM [25-27]. The BIA equation developed in this study (R^2 ^= 0.97; SRMSE= 1.99) was similar to or better than those obtained with other equations designed with comparable sample sizes, such as that of Lukasky (84 males; 67 females) [27] based on hydrodensitometry (R^2 ^= 0.96; SEE = 3.06), or of Baumgartner (35 males; 63 females)[28] based on a four compartment model (R^2 ^= 0.91; SEE = 2.47).

There were no differences between the mean predicted FFM and measured values by ADP. The pure error was small and comparable to good cross-validated equations reported in the literature [15]. There is no criterion value for the pure error that indicates successful cross-validation. However, the pure error should be similar to the precision (square root of the MSE value, SRMSE) of the validated equation in the population from where it is derived. Here the pure error is slightly higher than the SRMSE, indicating that the cross-validation was successful.

The new equation showed good precision. Similar findings were reported by Rising et al. [8]; the specific BIA equation developed (using hydrostatic weighing) for Pima Indians improved the average accuracy to -0.1 ± 3.3 kg versus the manufacturer's equation, which underestimated the fat-free mass by 5.3 ± 8.6 kg. Other studies have shown poor validity when equations designed and validated in one population have been used to predict FFM in other populations [29].

One of the most important justifications for designing and validating BIA estimation equations for specific groups is their differences in limb length [30]. The explanation for this could be that limb length and body weight are positively related to resistance, which is the main predictor of FFM estimated with BIA [27]. Limb length and body weight are strongly related to ethnicity and environmental factors. Deurenberg et al. demonstrated that limb length, expressed as leg length/height, influences the body composition values obtained with BIA; the length of the limbs was different among Chinese, Malays, and Indians. The authors consider the bias between TBW obtained with the deuterium dilution method and BIA values to be due to ethnic differences [30].

The regression analysis between FFM values estimated with the BIA equation validated in the present study and ADP (Figure 1) showed that the BIA equation was accurate and precise. Furthermore, Bland and Altman analysis determined there was no significant bias for FFM values obtained with the validated equation (Figure 2). These results are those expected for an equation that is population specific.

A recent and thorough review in the assessment of body composition using ADP[6] analyzed the validity of ADP relative to hydrostatic weighing (HW) in male and female adults, age 20–56 years. Mean group differences between ADP and HW measurements ranged from -4% to 1.9 % body fat; 5 of the 12 studies showed no significant difference between the two methods. Of the other 7 studies that did show a significant mean difference, the direction of the differences was inconsistent. Percent fat measured by ADP explained 78–94 % of the variance in % body fat measured by HW and the reported SEEs ranged from 1.8 to 2.3 % and ideally the range should be < 2.5 % body fat. These authors state that, whether the sex of the subject systematically affects results from ADP or HW remains to be determined. Because males tend to be leaner than females, it is difficult to determine whether the significant effect of sex reported in some studies are due to an effect of sex per se or to body fatness. Plotting the results from different studies as sex-specific means, in Bland-Altman fashion, they observed an upward trend with no overlap in mean % body fat between females and males. Therefore they conclude that in their analysis as in the individual studies, it is impossible to separate the confounding effects of subject sex and % body fat. Even though many studies consider ADP as a reliable and valid technique applicable in a wide range of subjects including children and elderly, more validation studies are required using a 4 Compartment model as a reference gold standard rather than HW. Other studies [31] suggest that that it is important to test its accuracy in more heterogeneous samples, or in individuals outside the average % fat range, specifically lean individuals, and insist in looking at possible gender bias. In our laboratory we have previously validated ADP using a 4 Compartment model in elderly subjects [32] as well as in children and adolescents (studies in progress).

## Conclusion

The new bioelectrical impedance equation based on a two-component model method was accurate, precise, and free of bias. The prediction validated equation can be used to assess body composition and nutritional status in populations having anthropometric and physical characteristics similar to this sample. However, we acknowledge the fact that there might be a problem of generality because it does not include Mexicans from all the country. Ethnic and environmental differences are the main factors that could affect the general applicability of the equation, and it should be used with caution.
